# The effectiveness of agomelatine on headache severity and frequency in episodic migraine without aura; a parallel randomized controlled trial study

**DOI:** 10.1186/s12883-023-03516-9

**Published:** 2024-01-02

**Authors:** Kourosh Farzin, Azita Kheiltash, Abbas Tafakhori, Nasim Ebadati Nakhjiri, Mahdi Shafiee Sabet, Nahid Dehghan Nayeri

**Affiliations:** 1 Department of Family Medicine, School of Medicine, Ziaeian Hospital, Tehran University of Medical Sciences, Tehran, Iran; 2 Department of Community Medicine, School of Medicine, Tehran University of Medical Sciences, Tehran, Iran; 3 Department of Neurology, School of Medicine, Iranian Center of Neurological Research Neuroscience Institute, Imam Khomeini Hospital Complex, Tehran University of Medical Sciences, Tehran, Iran; 4 Nursing Education Nursing and Midwifery Care Research Center, School of Nursing & Midwifery, Tehran University of Medical Sciences, Tehran, Iran

**Keywords:** Migraine, Agomelatine, Headache severity, Headache frequency

## Abstract

**Background:**

Migraine is a headache disorder that affects public health and reduces the patient’s quality of life. Preventive medication is necessary to prevent acute attacks and medication overuse headaches (MOH). Agomelatine is a melatonin antagonist.

**Aims:**

This study aimed to determine the effectiveness of agomelatine on the severity and frequency of migraine attacks.

**Methods:**

The study is a parallel randomized controlled trial with two groups of intervention and control. 400 patients were evaluated. Eligible individuals, including those with episodic migraine headaches without aura between the ages of 18 and 60 years who did not receive preventive treatment beforehand, were enrolled. Also, patients did not receive any specific medications for other diseases. Among these, 100 people met the inclusion criteria and entered the study. These subjects were randomly assigned to one of the two groups. The intervention group received 25 mg of agomelatine daily and the control group received B1. In this study, the effect of agomelatine on the frequency and severity of attacks, mean monthly migraine days (MMD), and migraine disability assessment (MIDAS), were assessed. The study was triple-blind and after three months, a post-test was performed. Data were analyzed using SPSS software.

**Results:**

A total of 100 patients were randomly assigned to either intervention or control groups. The prescriber physician and the data collector did not know about the allocation of patients to groups. Before the intervention, there was no significant difference in the headache frequency per month (t=-0.182, df = 98, *p* = 0.85), mean MMD (*p* = 0.17), headache severity (*p* = 0.076), and MIDAS (*p* = 0.091). After the study, there was a significant difference between the two groups in terms of the headache frequency per month (*p* = 0.009), and mean of MMD (*p* = 0.025). There was also a significant difference between pretest and posttest in two groups in the headache severity (*p* < 0.001) and MIDAS (*p* < 0.001).

**Conclusion:**

Agomelatine can be used as a preventive medication for migraine without aura. It is suggested that agomelatine be studied in comparison with other preventive drugs for patients with migraine.

**Trial retrospectively registration:**

Trial Retrospectively registration= IRCT20230303057599N1. Date: 2023-5-24 The present study is a residency thesis approved by the Tehran University of Medical Sciences.

## Introduction

Migraine is one of the 200 headache disorders [1]. This common neurovascular disorder is characterized by recurring headaches ranging from mild to severe headaches. It is the second most prevalent debilitating disease [2, 3], and affects over one billion people worldwide [4, 5]. Migraines are often accompanied by neck pain, muscle tension, sensitivity to light and sound (photophobia, and phonophobia), and symptoms in the autonomic nervous system like nausea and vomiting [5].

The reported prevalence of migraines varies among regions and populations [2]. It also varies in various texts and sources, with estimates ranging from about 1 to 2% of the general population, up to 14 to 15%. Some studies have shown that the prevalence of migraine is 18% in women and 6% in men [1, 2, 5–8]. However, comprehensive statistics regarding the prevalence of migraine in Iran are lacking. The highest reported prevalence of migraine in articles is in Tehran at 18.11% [5]. This widespread occurrence and the associated disabilities have a range of negative effects on patients, their families, colleagues, and society [4].

The etiology of migraine is not fully known [5], and many patients go undiagnosed and untreated [3]. An untreated migraine attack can last for 4 h, and in some cases, 2 or 3 days [9]. Due to the high prevalence and complications associated with migraines, as well as the resulting loss of productivity and quality of life [10, 11], it is crucial to address this condition and closely focus on its treatment. As a result, numerous researchers continue to conduct studies to investigate the effects of various medications on migraines. Therapeutic strategies primarily target acute and preventive treatment. Acute migraine treatment aims to provide pain relief within two hours of taking the medication (significant pain relief is also acceptable). Additionally, a sustained response for 24 h with minimal or no side effects, relief of accompanying symptoms (such as phonophobia, photophobia, nausea, and vomiting), and the ability to resume daily activities should be considered. The goal of preventive migraine treatment is to achieve at least a 50% reduction in mean MMD for episodic migraines and at least 30% for chronic migraines [12]. Preventive therapy is necessary to decrease the frequency and severity of headaches and also to prevent the occurrence of Medication overuse headache (MOH) [13, 14].

Although different guidelines on acute and preventive treatments have been developed and presented in the National Institute for Health and Care Excellence (NICE), these are mostly based on the medicines available in the country of origin. Additionally, some references have noted that treatments are sometimes ineffective or not tolerated by the patients [3, 12, 15, 16]. Therefore, there appears to be no consensus on the choice of medication at present.

However, studies investigating the different mechanisms of a migraine attack emphasize the role of various neuropeptides and neurochemical systems, such as melatonin. Melatonin shows promise as a treatment for migraines due to its lack of significant side effects and pharmacological interactions [17]. The results of a study on the levels of serotonin (ST) and melatonin (MT) hormones in the blood of 45 migraine patients and 35 healthy individuals showed that migraine patients had lower levels of serotonin (ST) and melatonin (MT) hormones compared to the control group [18].

Agomelatine belongs to the melatonin agonist class and has been studied in various research areas, including depression disorders [19], nephrotoxicity caused by paracetamol [20], pain threshold and neurogenesis [21], and depressive episodes in pregnancy [22]. Some studies have also examined the effects of melatonin and agomelatine on migraines. For example, a case series study was conducted on six patients with both depression and migraines. These patients had previously been treated with preventive drugs (such as amitriptyline, beta-blockers, and topiramate) for their headaches but did not respond to the treatment. However, when they were treated with agomelatine, a significant reduction in both depression and migraine symptoms was observed [23]. Additionally, other case studies have reported instances of migraine recovery in individuals [24]. Also, a randomized, double-blind trial in adolescents evaluated the effectiveness of melatonin in migraine prevention and showed a decrease in the frequency of migraines in this group [25]. A prospective comparative study with 200 patients with migraines in Iraq showed that melatonin is effective, if not superior to topiramate, for episodic migraine prophylaxis. Additionally, melatonin is better tolerated and has fewer adverse events than topiramate [26]. In a randomized, parallel, one-sided clinical trial conducted in Yazd, Iran, the effectiveness and safety of amitriptyline and melatonin for pediatric migraine prophylaxis were compared over a period of three consecutive months. The study concluded that both amitriptyline and melatonin are effective and safe for this purpose [27]. A randomized, double-blind, placebo-controlled study on women aged 18–65 years with migraine with or without aura was revealed that Melatonin is better than placebo for migraine prevention, more tolerable than amitriptyline and as effective as amitriptyline [28].

Also, systematic reviews and meta-analyses have found that melatonin may be beneficial in preventing migraines in adults [29, 30]. Another systematic review and meta-analysis for migraine prevention in adults showed that melatonin can significantly reduce the frequency and duration of migraine attacks, as well as the use of analgesics and the severity of migraines [31]. Additionally, a systematic review and meta-analysis of 25 clinical trials involving 4499 patients found that oral melatonin was associated with the most significant improvement in migraine frequency [32]. However, these studies noted that while melatonin has shown promise for episodic migraine prevention, there is no conclusive evidence for its effectiveness and they recommended further high-quality randomized controlled trials to provide complementary evidence [29, 32].

Therefore, due to the lack of consensus on the choice of medication in clinical guidelines as well as the mechanism of agomelatine effect, and based on the recommendations of some aforementioned studies, the researchers conducted the present study. The aim was to determine the effectiveness of agomelatine as a preventive therapy for patients with episodic migraine. The study focused on evaluating its impact on headache severity and frequency, MIDAS, and mean MM in patients referred to the major headache clinics of Tehran University of Medical Sciences.

## Materials and methods

This study was a Parallel randomized controlled trial (RCT) with a pretest, post-test, and a waiting control group. Patients who met the inclusion criteria were randomly assigned to one of the intervention or control groups using a random number table. Inclusion criteria were patients with episodic migraine headaches without aura, between the ages of 18 and 60 years’ old who had been referred for headaches and were not receiving preventive treatment before. Also, patients with other chronic diseases such as cardiovascular, liver disease, high blood pressure or diabetes were not included in this study. Additionally, patients who did not have any known mental disorders were chosen for the study.

Initially, 400 patients with migraines were evaluated in two big general hospitals affiliated with Tehran University of Medical Sciences. Among these, 100 patients were enrolled based on the calculation of sample size in interventional studies, according to previous research and drug effect size with the alpha of 5% and beta of 20%. Patients were enrolled in the study sequentially, based on the time of admission after filling out the informed consent form. After random allocation and before the intervention, all samples were evaluated in terms of severity and frequency of headaches, the number of headache days per month as well as MIDAS. To preserve the blinding of research samples, the control group was given vitamin B1 tablets with the same form and design of agomelatine. The B1 was chosen based on the following reasons: firstly, it is not listed among the known medicines for migraine relief in the guides related to NICE and the USA Headache Society. Additionally, this vitamin is soluble in water, easily excreted from the body, and does not have harmful effects. Furthermore, after consulting with the pharmaceutical factory, we selected this drug because it can be easily manufactured in the form of agomelatin, resembling it in appearance and consistency.

In this way, agomelatine and vitamin B1 were labeled as A and B, and only the principal investigator (M.S.) was aware of the type of medicines. The prescriber physician (K.F.) did not know about the assignment of individuals to groups so that recommendations and training were given equally. Patients in both groups were instructed to take pain-relieving medications (Acetaminophen or NSAIDs to triptans) at the onset of headaches and follow the recommendations for migraine prevention, including correcting sleep and time, modifying diet, and eliminating headache triggers. In addition to acute migraine treatment, the intervention group received 25 mg of agomelatine daily as a preventive treatment and the control group received a B_1_ Tablet. The intervention was performed for each patient for three months and then a post-test was performed. The duration of the enrollment of the samples was six months until the completion of the study. According to the results, agomelatine was started for the control group after the post-test.

**Fig. 1:**
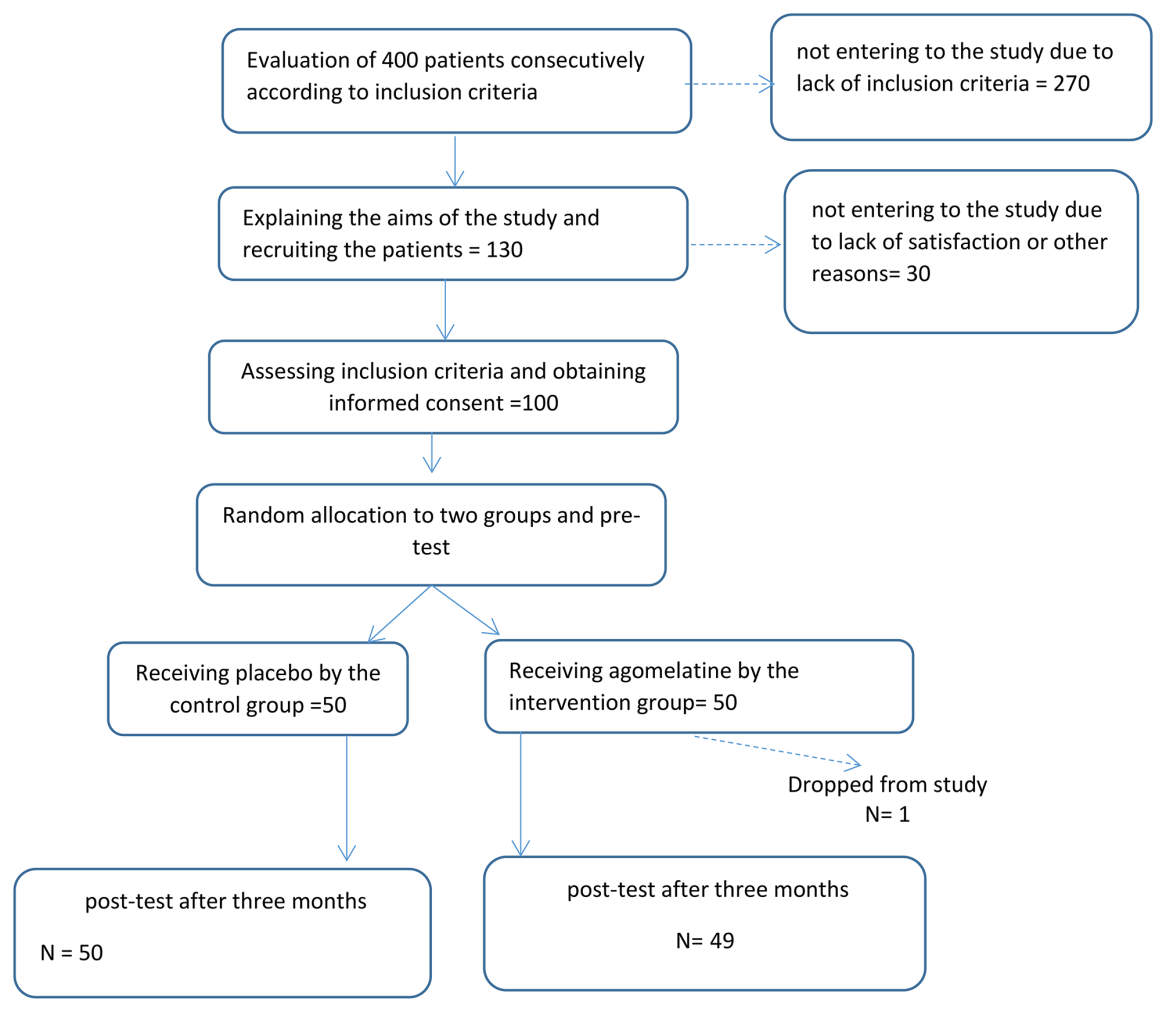
CONSORT diagram

The data were collected using the questionnaires (frequency and severity of headache, the number of headache days per month, and MIDAS), which were filled by the research colleague by interviewing the patient. Data were analyzed by SPSS software version 16 and descriptive and inferential statistics such as mean, standard deviation, and paired and independent t-tests. The data analyst was not aware of group allocation until the end. To analyze the demographic data (Table 1), we used the t-test to compare two groups (such as age) if the data were continuous and met the assumptions of parametric statistics. However, if the data were classified (such as gender, marital status, education degree), we used the chi-square test to compare the two groups. In cases where the assumptions of the chi-square test were not met, we used Fisher’s statistical test, as was done for occupational status. Since the assumptions of parametric statistics were met, independent and paired t-tests were used to compare pre-test and post-test data on the main variables (Tables 2 and 3, and 4). Figure 1 which is the CONSORT diagram shows the stages of the study.

## Results

A total of 50 patients were enrolled in the control group and 50 in the intervention group, of which 99 people completed the study. There was no significant difference in demographic data of the two groups including age, sex, marital status, educational degree and occupational Status. Table 1 shows the demographic characteristics of the two groups. Also, there was no significant relationship between demographic variables and other dependent variables (*p* > 0.05). Analysis of variance showed no significant relationship between marital, educational, and occupational status and dependent variables (*p* > 0.05). The only significant difference was in headache severity between males and females before the intervention, shown in the independent t-test (t = 2.58, df = 98, *p* = 0.011).

**Table 1:** Demographic characteristics of patients with migraine in two groups

Variables		intervention		control		Results
		frequency	percent	frequency	percent	
Age	< 25	3	6	10	20	*p* = 0.091
	26–35	18	36	18	36	
	36–45	17	34	13	26	
	46–55	10	20	8	16	
	> 55	2	4	1	2	
Sex	male	39	78.0	36	72.0	*p* = 0.49
	female	11	22.0	14	28.0	
Marital status	single	43	86.0	41	82.0	*p* = 0.54
	married	7	14.0	8	16.0	
	divorced	0	0	1	2.0	
Educational degree	Less than a diploma	22	44.0	18	36.0	*p* = 0.62
	diploma	14	28.0	16	32.0	
	Associate Degree	4	8.0	2	4.0	
	Bachelor student	3	6.0	1	2.0	
	Bachelor’s degree	5	10.0	8	16.0	
	Master’s degree	2	4.0	5	10.0	
Occupational status	housewife/retired	33	66	25	50	*P* = 0.34
	outsider job	2	4	5	10	
	employee	6	12	12	24	
	self-employed	9	18	8	16	
Total		50	100	50	100	

Before intervention the differences between control and intervention groups in terms of the attacks in the previous month, the average number of headache days, the severity of headache, and MIDAS were not significant (Table 2).

**Table 2:** Comparison of outcome variables in the pretest of intervention and control groups

Variable	Pretest				Results
	intervention		control		
	Mean	SD	Mean	SD	
headache attacks in the previous month	6.16	1.65	6.10	1.34	*p* = 0.86
average number of headache days	11.94	3.54	11.06	2.78	*p* = 0.172
severity of headache	7.43	1.1	7.04	1.07	*p* = 0.076
Midas	19.06	5.31	17.47	3.85	*p* = 0.091

Three months after the intervention, there was a significant difference between the control and intervention groups in terms of the number of headache days per month and the headache attacks. Also, the above two groups had significant differences in terms of MIDAS changes and headache severity from the pre-test to the post-test (Table 3).

Also, the paired t-test showed a significant difference in the post-test compared to the pretest in the intervention group in all four mentioned outcomes (*p* < 0.05) (Table 4). However, in the control group, only the difference in the headache severity in the post-test compared to the pre-test was significant (Table 5).

**Table 3:** Comparison of outcome variables in the post-test after three months of intervention and control groups

Variable	Posttest after three month				Results
	intervention		control		
	Mean	SD	Mean	SD	
headache attacks in the previous month	4.8	2.2	5.82	1.5	*p* = 0.009
average number of headache days	8.86	4.5	10.63	3.1	*p* = 0.025
Change in headache severity	-4.1	3.4	-0.71	2.6	*p* < 0.001
Midas change	-1.06	0.9	-0.36	0.63	*p* < 0.001

**Table 4:** Comparison of outcome variables in the post-test with pretest of intervention groups

Variables	Intervention group				Results
	Pretest		posttest		
	Mean	SD	Mean	SD	
headache attacks in the previous month	6.16	1.65	4.8	2.2	*p* < 0.001
average number of headache days	11.94	3.54	8.86	4.5	*p* < 0.001
headache severity	7.43	1.1	6.35	1.2	*p* < 0.001
Midas	19.06	5.31	15.04	6.6	*p* < 0.001

**Table 5:** Comparison of outcome variables in the post-test with pretest of control group

Variables	Intervention group				Results
	Pretest		posttest		
	Mean	SD	Mean	SD	
headache attacks in the previous month	6.10	1.34	5.82	1.5	*p* = 0.061
average number of headache days	11.06	2.78	10.63	3.1	*p* = 0.13
headache severity	7.04	1.07	6.67	1.1	*p* < 0.001
Midas	17.47	3.85	16.76	4.28	*p* = 0.065

## Discussion

The majority of patients in both groups were female, which is consistent with the prevalence mentioned in several articles [7, 33, 34]. The findings showed no significant difference between the headache attacks and the average number of headache days per month between the two groups. The number of attacks per month was slightly more than 6 times in both groups. In a systematic review study, the number of attacks before treatment was 4.2 [30], lower than the number of attacks in the current study, which could be due to demographic variables of the participants.

The mean MMD was not significantly different in the two groups (nearly 11 days in the intervention group and 12 days in the control group). These findings are almost consistent with other studies. In one study, the mean MMD and monthly headache days (MHD) were respectively 10.37 and 22.24 [35]. In another study with 1072 participants mean MMD was approximately 16.1 at baseline [36]. In the current study, the number ranged between 11 and 12, which falls within the range mentioned in previous studies.

Results showed no significant difference between the pretest and posttest regarding the frequency of attacks (*p* = 0.061), mean MMD (*p* = 0.13), and MIDAS (*p* = 0.065) in the control group. However, there was a significant difference between headache severity in the control group before and after intervention (*p* < 0.001). Several studies show that some B vitamins can improve migraine headaches, especially frequency and severity. Dietary supplements B vitamins can effectively prevent or reduce the various symptoms of migraine headaches [37]. Also, in other studies, there was a decrease in mean MMD in the placebo-treated group, which varied depending on the route of medication, the type of migraine, and the effect of previous treatments [38].

However, the findings for the intervention group showed significant differences before and after treatment, in all four variables including the headache attacks per month, the mean MMD, headache severity, and MIDAS. The headache attacks decreased significantly after the test, with the average decreasing from 6.16 to 4.8. Additionally, the average number of headache days in a month decreased from 11.94 to 8.8, which is a significant result. The mean severity also decreased from 7.43 to 6.45 which is a significant result. MIDS was another variable that decreased significantly.

Additionally, these findings revealed significant differences in the posttest results between the intervention and control groups. This suggests that agomelatine is effective as a prophylactic medication for patients with episodic migraine without aura who are not currently using preventive medicine. Other studies have also reached results that are in line with the findings of this article, including case series studies, clinical trials and systematic reviews of RCTS [23–26, 28]. However, achieving a 50% reduction in migraine attacks or their severity is considered as a criterion in the clinical therapy and extensive research is necessary to meet this criterion. According to the data analysis in this study, many patients in the intervention group successfully met this criterion; but there are still individuals who have not reached this point. Nonetheless, it is crucial to publish research that provides valid evidence of effectiveness. Relying solely on studies with high effectiveness can introduce publication bias for producing the evidence [39]. It is reasonable that further research is required to investigate specific doses and combinations with other drugs in the group who experience higher attacks.

In patients with migraine, low levels of melatonin in serum and urine have been reported, due to hypothalamic dysfunction; therefore, melatonin can be considered a safe preventive treatment for migraines. Research on melatonin administration in migraine patients has shown it to be safe with few or no side effects. Melatonin plays an important role in regulating circadian rhythm and sleep-wake cycles [40]. The systematic study reports that according to data from controlled and uncontrolled studies, there is a strong relationship between melatonin and headache relief. In observational studies, migraines were significantly improved compared to baseline [30]. On the other hand, observational studies support the effectiveness of melatonin in migraine. In conclusion, melatonin is likely to be beneficial for migraine prevention and may have the same effectiveness as other major preventive medications. Melatonin plays a role in the opioid system, nitric oxide pathway, free radical removal, and inflammation. The effectiveness of melatonin in migraine prevention is growing in current literature but is still limited [30, 41].

## Conclusion

According to the current study, agomelatine can be used to reduce the number of attacks, mean MMD, MIDAS, and headache severity. Because this study was done on episodic migraines and the control group received a B_1_, the researchers suggest comparing agomelatine with other medications to develop a body of knowledge in this area.

## Data Availability

If someone wants the data, they can request it from Mahdi Shafiee Sabet, the corresponding author of the article. Currently, the data is not published publicly, but if the journal requests it, we will publish it publicly.
